# Large-scale fabrication of boron nitride nanotubes with high purity via solid-state reaction method

**DOI:** 10.1186/1556-276X-9-555

**Published:** 2014-10-07

**Authors:** An Pan, Yongjun Chen

**Affiliations:** 1 College of Chemistry & Chemical Engineering, Guangxi University, Nanning 530004, China; 2 Key Lab of Advanced Materials of Tropical Island Resources, Ministry of Education, College of Materials and Chemical Engineering, Hainan University, Haikou 570228, China

**Keywords:** Boron nitride nanotubes, Fabrication, Photoluminescence property, Growth mechanism

## Abstract

An effective solid-state reaction method is reported for synthesizing boron nitride nanotubes (BNNTs) in large scale and with high purity by annealing amorphous boron powder and ferric chloride (FeCl_3_) catalyst in ammonia atmosphere at elevated temperatures. FeCl_3_ that has rarely been utilized before is introduced not only as a catalyst but also as an efficient transforming agent which converts boron powder into boron chloride (BCl_3_) vapor in situ. The nanotubes are bamboo in shape and have an average diameter of about 90 nm. The effect of synthetic temperatures on nanotube morphology and yield is investigated. The photoluminescence (PL) measurement shows emission bands of the nanotubes at 354, 423, 467, and 666 nm. A combined growth mechanism of vapor–liquid-solid (VLS) and solid–liquid-solid (SLS) model is proposed for the formation of the BNNTs.

## Background

As an isostructural and isoelectronic analog to carbon nanotubes (CNTs), boron nitride nanotubes (BNNTs) have attracted more and more attention on account of their superb mechanical property [1], outstanding thermal conductivity and stability [2], excellent radiation shielding [3], strong light emissions [4], and hydrogen storage capacity especially the bamboo-shaped BNNTs [5]. In addition, recent theory simulations reveal that metal-doped (e.g., Fe, Al) BNNTs seem to be more sensitive to some specific absorbates [6,7]. BNNTs are not cytotoxic and can be functionalized for interaction with proteins and cells [8]. Partially vertically aligned BNNT films deposited on Si substrate exhibit strong water repellency [9]. All these fascinating properties make BNNTs promising applications in many areas such as lasing action, gas detective sensors, nanocoating for composites, reinforcement additive [10], hydrogen storage devices [11], and biomedical materials.

Since the discovery of BNNTs in 1995 [12], the main approaches known for the growth of CNTs have been modified to synthesize BNNTs as well, for instance, arc discharge [13], laser ablation [14], and chemical vapor deposition [5]. Unfortunately, however, the yield or purity of BNNTs prepared by such methods is normally disappointing in comparison with that of CNTs. The demands of property investigation and applications are required for an effective route to mass production of BNNTs with high purity. Chen et al. [15] developed a ball milling and annealing method as potential for large-scale production of BNNTs, which gave a conception that a solid-state reaction method would be an alternative technique in bulk synthesis of BNNTs. Nonetheless, particles usually coexisted with BNNTs in the product, which needed further purification process before application. In the current study, we report an effective solid-state reaction method which offers BNNTs with both large quantity and high purity by annealing a highly homogenous precursor composed of amorphous boron (B) powder and ferric chloride (FeCl_3_) in flowing ammonia (NH_3_) atmosphere. To the best of our knowledge, FeCl_3_ has never been employed as the catalyst for the synthesis of BNNTs. Furthermore, FeCl_3_ could also act as a crucial transforming agent that converted solid-state B powders to gaseous boron chloride (BCl_3_) in situ. The gaseous BCl_3_ possessed high reaction activity and guaranteed the formation of BNNTs under NH_3_ flow. The effect of synthetic temperature on the morphology and the growth mechanism of the BNNTs was discussed in detail. The photoluminescence (PL) property of the synthesized BNNTs was also investigated.

## Methods

### Synthesis of BNNTs

In a typical procedure, amorphous B powders and FeCl_3_ · 6H_2_O with a molar ratio of 1:0.05 were selected as the raw materials. Firstly, FeCl_3_ · 6H_2_O was dissolved in absolute ethyl alcohol, then B powders were added into the solution. The mixture was stirred and heated in the water bath at 40°C for 2 h in order to evaporate the solvent. Then the obtained paste-like mixture was dried at 55°C to thoroughly remove the ethanol. After, a highly homogeneous distributed precursor was prepared. The precursor was loaded into an alumina boat which was placed at the center of a tube furnace. Before heating up, high-purity NH_3_ flow was introduced to flush out the residual air in the chamber. Then the furnace was heated to 1,200°C at 50 mL min^−1^ NH_3_ flow and maintained for 5 h. Finally, the furnace was naturally cooled to ambient temperature under the protection of N_2_ flow.

### Characterization of BNNTs

Afterwards, it was found that the color of the upper layered staring materials changed from brown into white; the white product was characterized to be BN nanosheet self-assembled microwires and will be discussed elsewhere. Beneath the white product, a gray product was obtained in the boat. The gray product was collected and characterized by scanning electron microscopy (SEM, Zeiss Merlin; Carl Zeiss AG, Oberkochen, Germany), transmission electron microscopy (TEM) and high-resolution TEM (HRTEM, JEOL JEM-2011; JEOL Ltd., Tokyo, Japan) equipped with X-ray energy dispersive spectrometer (EDS), and electron energy loss spectroscopy (EELS, FEI Titan 80–300; FEI, Hillsboro, OR, USA). The PL property of the product was measured at room temperature (Edinburgh FLS920, 300-nm excitation; Edinburgh Instruments, Hertfordshire, UK).

## Results and discussion

**Figure 1:**
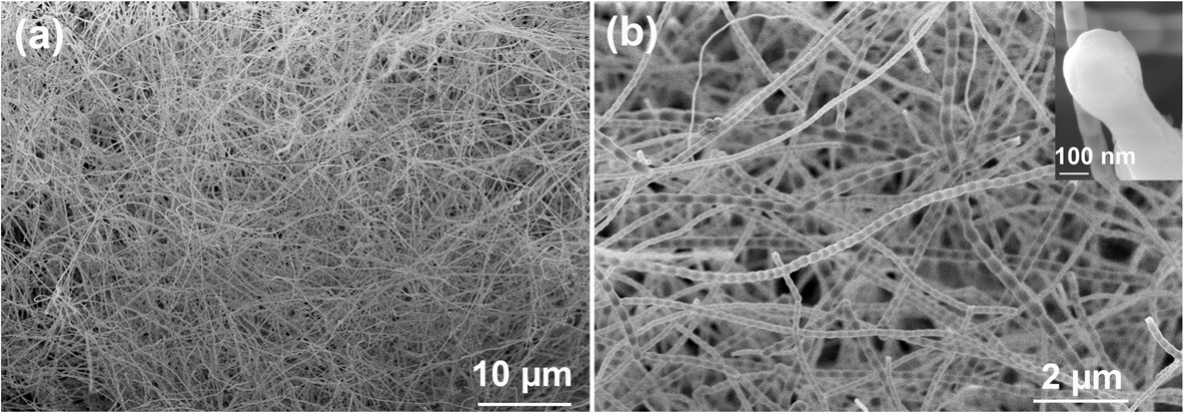
**SEM images of the product synthesized at 1,200°C. (a)** Low-magnification image. **(b)** High-magnification images. The inset shows a particle attached at the end of a nanotube.

The SEM images of the as-synthesized sample are shown in Figure 1. Low-magnification image (Figure 1a) exhibits that a large quantity of one-dimensional (1D) nanostructures is synthesized. The nanostructures are dense and relatively pure (no obvious particles can be observed). Figure 1b shows the high-magnification image of the nanostructures. It reveals clearly that the nanostructures are bamboo in shape (i.e., nanotubes) with smooth surfaces. The diameters of the nanotubes vary from 20 nm to more than 100 nm with a mean value of about 90 nm. Further magnified image (the inset) shows a particle attached at the end of a nanotube, which is normally regarded as a typical symbol of vapor–liquid-solid (VLS) growth model.

**Figure 2:**
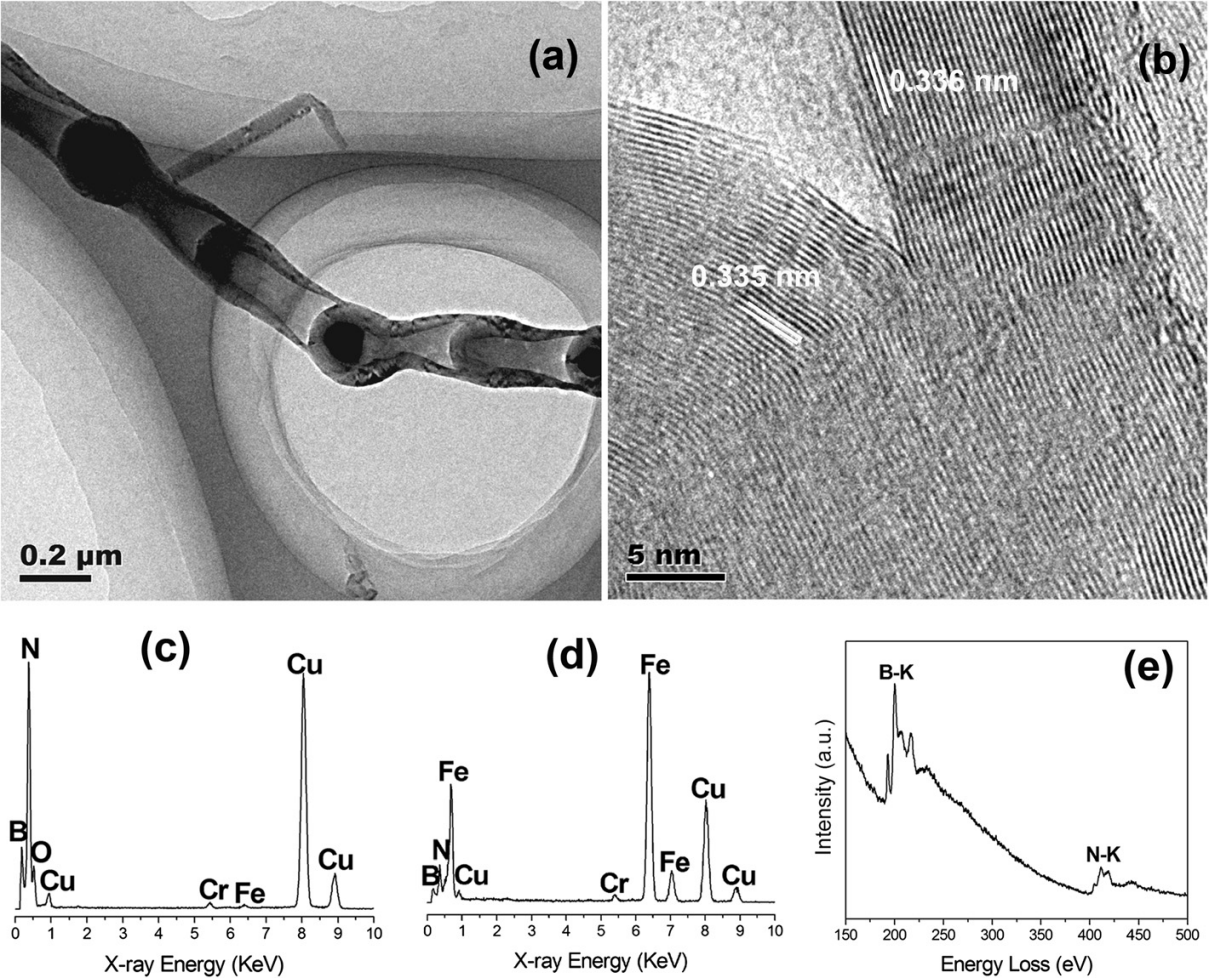
**TEM images and composition of the synthesized BNNTs. (a)** TEM image. **(b)** HRTEM image. **(c)** EDS result of the nanotube walls. **(d)** EDS result of the encapsulated particles within the nanotube. **(e)** EELS spectrum of the nanotube.

The TEM image shown in Figure 2a illustrates more clearly that the nanostructure is a bamboo-shaped nanotube composed of a series of hermetical compartments that are tightly connect together. At the same time, catalyst particles are observed within the cavity of the nanotube, which is consistent with the SEM observation. Figure 2b demonstrates the HRTEM image of a joint connecting the wall and the compartment of the nanotube. The lattice fringes of the wall and compartment can be clearly seen with interlayer spacings of approximately 0.336 and 0.335 nm, respectively, which correspond to (002) planes of hexagonal BN (h-BN) crystals. The slight difference of interlayer spacings between the wall and compartment can be attributed to the measurement error. Figure 2c is the EDS spectrum taken from the nanotube walls, demonstrating major peaks of B, N, and Cu with small amounts of O, Fe, and Cr. The Cu peaks should come from the copper TEM grid, while O peak is ascribed to the slight surface oxidation or oxygen adsorption of the nanotube in air. The weak Fe peaks should be caused by the inclusion of FeCl_3_. We speculate that Cr signal may come from the impurities in the raw materials. Therefore, it can be roughly concluded that the nanotube is composed of BN. Figure 2d shows the EDS result of an encapsulated particle. Dominating peaks of B, N, Fe, and Cu along with a low level of Cr are detected. Apparently, the intensity of Fe signal increases dramatically. It is therefore believed that Fe-containing alloy droplets exist historically during the growth of BNNTs. The chemical composition of the nanotube is further determined by means of EELS. Figure 2e depicts a typical EELS spectrum that has two pronounced adsorption peaks of B and N characteristic K-edges at 188 and 401 eV, respectively. For each K-edge adsorption, a discernible π^*^ peak accompanied by a broad σ* peak can be observed, which is a representative feature of an sp^2^-hybridized state. Hence, it is confirmed that the synthesized product comprised bamboo-shaped BNNTs.

**Figure 3:**
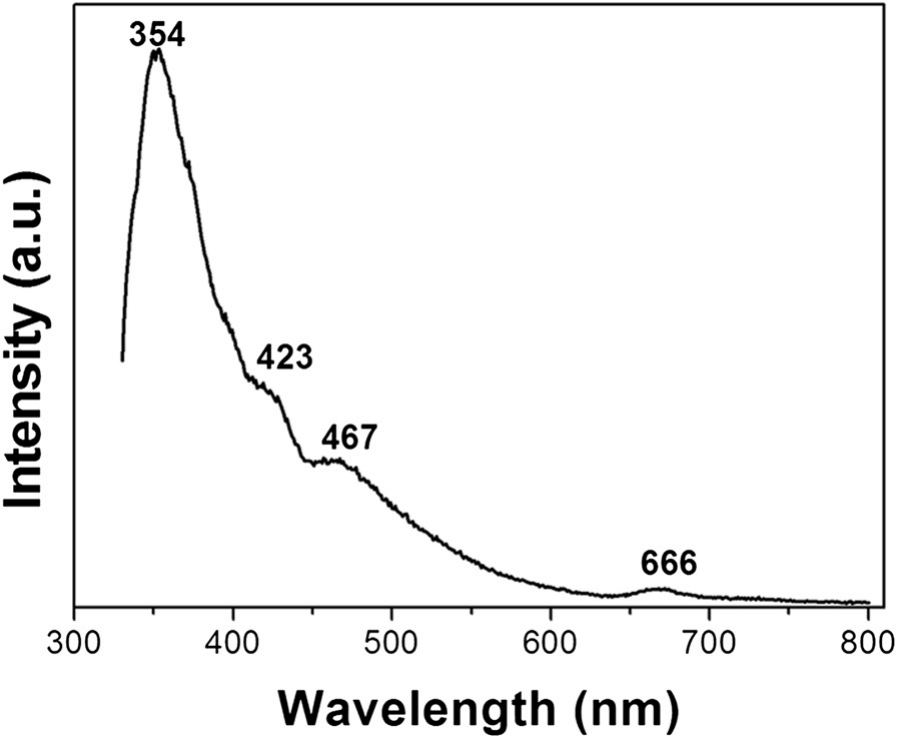
Photoluminescence property of the synthesized BNNTs.

**Figure 4:**
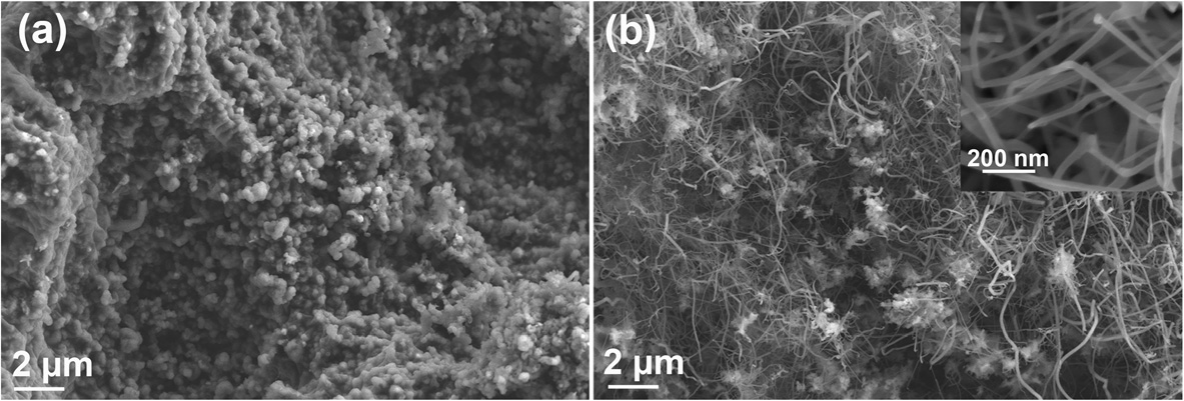
**SEM images of the products synthesized at different temperatures. (a)** 1,150°C. **(b)** 1,250°C.

Figure 3 shows the PL property of the BNNTs. Four main emission bands centered at 354, 423, 467, and 666 nm are observed. Normally, the possibility of a material to demonstrate luminescence relies on the intrinsic band edge structure and other internal or external factors (intrinsic/extrinsic defects) [16,17]. Some PL emission peaks in the range 300 to 400 nm have been reported [18-21] that originate from residual impurities such as carbon and oxygen [22] rather than interband transitions. Therefore, the band at about 354 nm in our study could be assigned to the impurity centers (possibly attributed to oxygen impurities, as is supported by the EDS result). The emission bands located at 423 and 467 nm could be attributed to the intrinsic emission from bamboo-shaped BNNTs, which possess a great deal of bent layers and associated defects similar to the cup-shaped BN layers in the bamboo-shaped nanotubes [4]. There have been no correlated reports on the PL band located at 666 nm for h-BN. However, Zhu et al. stated that the emission band at 700 nm of BN whiskers might originate from defect-trapped states (vacancy-type defect) and a quantum confinement effect [23]. Long-wavelength PL emissions at 728 and 703 nm were reported by our group in BN nanowires [24] and nanotubes [25] due to the intrinsic lattice defects as well. Chen et al. [4] interpreted 680-nm PL emissions in periodic yard-glass-shaped BNNTs to be ascribed to the lattice defects in the periodical structures and their inserting connection mode. Therefore, it is reasonable that intrinsic lattice defects are responsible for the emission band at 666 nm in the current study.Figure 4a,b shows the SEM images of the product synthesized at 1,150 and 1,250°C, respectively. No nanotubes can be observed in the case of 1,150°C (Figure 4a). When the temperature increases to 1,250°C, the yield of BNNTs decreases obviously and considerable amounts of particles are formed in the product (Figure 4b). Moreover, careful observation from the enlarged image (the inset) reveals that the nanotubes are generally in the form of a quasi-cylindrical structure.

Based on the results described above, the growth process of the BNNTs is illustrated as follows:

(1) $$\begin{array}{l} 6FeCl_{3}\;\left(s\right)+\;2NH_{3}\to 6FeCl_{2}+\;\left(g\right)\;+N_{2}\;\left(g\right)\\ \;\Delta G<\;0\;\left(\mathrm{temperature}\;\mathrm{in}\;\mathrm{range}\;\mathrm{of}\;0∼1250^{o}C\right) \end{array}$$

(2) $$FeCl_{2}\left(s\right)\to FeCl_{2}\left(l\right)$$

(3) $$\begin{array}{l} FeCl_{2}\left(l\right)\to FeCl_{2}\left(g\right)\\ \;\Delta G\;\left(1150\;\sim 1250^{o}C\right)\;=\;-14.820\;\sim \;-24.670\;\mathrm{kJ} \end{array}$$

(4) $$\begin{array}{l} 3FeCl_{2}\left(g\right)\;+\;2B\;\left(s\right)\to 2BCl_{3}\left(g\right)\;+\;3Fe\;\left(l\right)\\ \;\Delta G\;\left(1150\;\sim 1250^{o}C\right)\;=\;‒44.265\;\sim \;‒22.991\;\mathrm{kJ} \end{array}$$

(5) $$\begin{array}{l} 3FeCl_{2}\left(l\right)\;+\;2B\;\left(s\right)\to 2BCl_{3}\left(g\right)\;+\;3Fe\;\left(l\right)\\ \;\Delta G\;\left(1150\;\sim 1250^{o}C\right)\;=\;‒88.726\;\sim \;‒97.000\;\mathrm{kJ} \end{array}$$

(6) $$\begin{array}{l} BCl_{3}\left(g\right)\;+\;NH_{3}\left(g\right)\to BN\;\left(s\right)\;+3HCl\;\left(g\right)\\ \;\Delta G\;\left(1150\;\sim 1250^{o}C\right)\;=\;‒216.077\;\sim \;‒226.045\;\mathrm{kJ} \end{array}$$

At first, FeCl_3_ is reduced to FeCl_2_ by NH_3_. Along with heating up, FeCl_2_ goes through a series of transformation from solid phase to liquid and gas phase (Equations 1, 2, and 3). Proved by the thermodynamic calculations, FeCl_2_ boils up mildly, and liquid-phase FeCl_2_ is more reactive than gas-phase FeCl_2_ in transforming solid B powders into BCl_3_ vapor at experimental temperatures (Equations 4 to 5). When the temperature reaches to 1150 to 1,250°C, Fe melts down partially and absorbs the surrounding vapors of BCl_3_ and NH_3_ to form Fe-B-N alloy droplets. When the concentrations of the BN species in the droplets are greater than the saturation threshold, BN shells begin to precipitate layer by layer around the droplets (Equation 6). The thickness of BN shell gradually increases, and the diameter of newly formed BN shell decreases with the inward growth of the BN shells. The increasing curvature caused by smaller diameter of the inner BN shell results in growing stress energy between the shells and the catalyst core [26]. When the stress energy reaches a certain degree, the molten core will be expulsed from the defective region of the BN shells and sequentially shrinks to locate at the door of the torn elongated BN shell. The expulsed catalyst core keeps on absorbing gas species and the next growth proceeds via the same way. The whole process is extremely similar to the VLS growth of bamboo-shaped BNNTs reported in literatures [27,28]. Particularly worth mentioning in the current study is that the catalyst core will be expulsed out wholly or partially depending on the value of stress energy, which can explain the TEM observation of discontinuous encapsulated particles within the compartment cavities of the nanotubes. As the described procedure occurs in cycles, bamboo-shaped structures are finally formed. It can be found that the growth process of the nanotubes not only involves the absorption and dissolution of NH_3_ and BCl_3_ from gas phase but also includes the relatively slow diffusion of B from solid phase to the alloy droplets, which may determine the final growth rate of the nanotubes. Therefore, the growth of BNNTs in the current study follows a combination mechanism of VLS and solid–liquid-solid (SLS) models [27,29].

When the temperature lowers to 1,150°C, the reactions (Equations 4 and 5) could still be carried out but the reaction rates reduce significantly. Moreover, the diffusion rate of B/N atoms on alloy droplet surfaces also decreases. Thus, the insufficiency of BCl_3_ vapor results in the formation of particles rather than nanotubes. As the reaction temperature rises to 1,250°C, both the diffusion rates of B/N atoms through the surface and the bulk of the catalyst droplets increase. However, the surface diffusion rate enhances significantly [30], which favors the formation of nanotube walls rather than the compartment layers. In addition, the crystalline perfection of the nanotubes improves with the rise of temperature and the compartment will endure stronger stress. Hence, a quasi-cylindrical structure with less strain will be more favorable based on the principle of minimum free energy. However, the nitridation reaction (Equation 6) performs very fast and the concentration of BCl_3_ vapor within the chamber increases so quickly within a short period of time that some of them will be flushed out of the chamber by NH_3_ flow. Therefore, the yield of the BNNTs diminishes.

## Conclusions

High-purity BNNTs are successfully synthesized in large quantity by annealing FeCl_3_ and amorphous boron in NH_3_ atmosphere. FeCl_3_ not only provides catalyst Fe but also reacts with boron powders to generate BCl_3_ vapor in situ, which is vital for the formation of BNNTs. The BNNTs are bamboo in shape with an average diameter of about 90 nm. A combination growth mechanism of VLS and SLS models is proposed to govern the formation of BNNTs. The synthetic temperature affects the morphology and yield of the BNNTs greatly through influencing the generation of BCl_3_ vapor. With the rise of temperature, the structure of the BNNTs has a tendency to form from bamboo toward cylindrical, but the yield of the BNNTs tends to be lower. The BNNTs exhibit strong PL emissions at 354 nm, suggesting their potential applications in optical and electronic devices.

## Competing interests

The authors declare that they have no competing interests.

## Authors’ contributions

AP performed the syntheses and characterization. YC was in charge of designing and supervision of the experiments. Both authors read and approved the final manuscript.
